# The relationship between phylogenetic groups and antibiotic susceptibility patterns of *Escherichia coli* strains isolated from feces and urine of patients with acute or recurrent urinary tract infection

**Published:** 2019-12

**Authors:** Hossein Norouzian, Mohammad Katouli, Nader Shahrokhi, Sharam Sabeti, Mohammad Pooya, Saeid Bouzari

**Affiliations:** 1 Department of Molecular Biology, Pasteur Institute of Iran, Tehran, Iran; 2 Genecology Research Centre, School of Health and Sport Sciences, University of the Sunshine Coast, Maroochydore DC, Queensland, Australia; 3 Deapartment of Pathology, Loghman Hakim Hospital, Shahid Beheshti University of Medical Sciences, Tehran, Iran

**Keywords:** Uropathogenic *Escherichia coli*, Acute urinary tract infection, Recurrent urinary tract infection, Antibiotic resistance, Phylogenetic groups

## Abstract

**Background and Objectives:**

B2 and D have been mentioned as the most common phylogenetic groups among uropathogenic *Escherichia coli.* However, there is still controversy about the importance of these phylo-groups. This study was conducted to investigate the probable relation between these groups and antibiotic resistance patterns of *E. coli* isolates derived from urine and feces of the patients with acute or recurrent UTI.

**Materials and Methods:**

10 isolates were recovered from urine and feces samples of patients with different phases of UTI in whom *E. coli* was causative pathogen. Biochemical fingerprinting was performed to classify the isolates and select their appropriate representatives. Phylogenetic grouping was performed using multiplex PCR, and antibiotic resistance was determined by disk diffusion method.

**Results:**

Five-hundred-sixty *E. coli* isolates were derived from 56 UTI patients (27 acute, 29 recurrent). Among them, 261 isolates were selected using biochemical fingerprinting. All the isolates were sensitive to imipenem and nitrofurantoin. Compared to other phylo-groups, the isolates in group D showed considerably different frequencies in acute vs. recurrent phase of UTI, in urine vs. stool samples, in males vs. females, and in- vs. out-patients. They were more resistant to the antibiotics (except norfloxacin), and in contrast to others, this was seen more in acute UTI, especially in urine samples. Multi-drug resistance pattern was also meaningfully higher in group D.

**Conclusion:**

Although phylo-groups B2 and D of *E. coli* bacteria are more responsible for UTI, group D isolates seem to be more resistant and probably more virulent, even than the ones from group B2.

## INTRODUCTION

Urinary tract infection (UTI) is undoubtedly one of the most prevalent infectious diseases, making it one of the globally health concerns, which predominantly affects women (1, 2). Over 25% of women with acute UTI experience recurrent infection usually within 6 months (3). Uropathogenic *Escherichia coli* (UPEC) are the most important causative bacteria; accounting for 65–75% of UTI cases, either acute or recurrent. For these UTI phases, two different pathogenesis pathways have been recommended so far. On one hand, it has been suggested that in both phases, contamination of the periurethra and colonization of the urethra with UPECs may lead to their migration to the bladder and develop UTI. On the other hand, it has been proposed that while in the acute phase, the pathogenesis may be as above; UPECs may settle on the immature urinary epithelial cells of the bladder, be activated during the maturity of these cells and act as a reservoir for the recurrent infection (4).

Epidemiological evaluation and classification of bacteria can be performed using several methods of typing such as serotyping and pulsed field gel electrophoresis (PFGE). Biochemical fingerprinting, a simple and automatic system for typing, has been used in many studies so far, especially in Enterobacteriaceae (5, 6). This method utilizes the differences in the reaction rates between isolates, in automatically identification based on biochemical phenotypes. The simplicity of data running and processing as well as high accuracy and easy analysis of data make it suitable for screening and classifying, especially for large number of isolates (6, 7). Meanwhile, it is similar to the PFGE in terms of discriminatory power and repeatability (8). In addition, this method has been previously used for evaluation of enterotoxigenic *E. coli* in humans and fecal colonization of *E. coli* in neonates with pyelonephritis (9).

According to phylogenetic grouping, *E. coli* strains can be divided into four main phylo-groups A, B1, B2 and D, which differ each other in characteristics such as antibiotic resistance profiles and colonization sites (10, 11). Analyzing *E. coli* phylo-groups around the world has shown that the extraintestinal pathogenic *E. coli* strains which carry more virulence factors belong to group B2, followed by group D, while most of the gut commensal strains are mainly from group A, and to lesser extent group B1 (12, 13).

Antimicrobial resistant gram-negative uropathogens such as UPEC has recently become a great concern. Studies have clearly demonstrated a greater resistance to antibiotics such as fluoroquinolones, third-generation cephalosporins, and carbapenems among them (14). Besides, multidrug resistant (MDR) Enterobacteriaceae, defined as “non-susceptible isolates to one or more agent(s) in three or more antimicrobial categories” (15), are increasing worldwide. In recent years, differences in antibacterial resistance of *E. coli* strains in certain phylogenetic groups have been considered (16). However, little studies have been done to discriminate these differences between acute and recurrent phases of UTI, urine and stool samples of patients or both.

The aim of this study was to determine and detect phylogenic groups and antibiotic resistance patterns of *E. coli* isolates obtained from urine and stool samples of the patients with acute or recurrent UTI, and to discover any probable relationship between them.

## MATERIALS AND METHODS

### Sample collection.

The present descriptive cross-sectional study was performed on *E. coli* strains isolated from the patients with either acute or recurrent UTIs during April 2016 to May 2017 in the Loghman Hospital, Tehran, Iran. This research was approved by the Ethics Committee of Research Center of Pasteur Institute of Iran. First, the fresh midstream urine specimens of any patient, either admitted or hospitalized, who had clinical symptoms of UTI (*i.e*. dysuria, frequency, and urgency, as well as fever and flank pain) were collected and cultured on MacConkey and Blood agar (Merck, Germany) media and incubated at 37°C for 24 hours. If the colony count was equal to or greater than 10^5^ CFU/ml, the urine culture was considered as positive and the patient was definitely assumed to have UTI. Then, the stool samples of these patients were obtained and cultured on Trypticase soy agar (Merck, Germany) with 5% sheep blood and MacConkey agar media. At the same time, a consent letter was signed by patients, and a questionnaire included required information such as age, gender, history of antibiotics consumption and history of any probable previous episodes of UTIs was completed by the investigator. The criteria for defining recurrent infection were “two separate culture-proven episodes of UTI and associated symptoms within six months or three episodes within one year” (17). Nevertheless, UTI episode was defined as acute infection.

All the samples were transported under sterile conditions to the Molecular Biology Department’s laboratory at Pasteur institute of Iran, where bacterial morphology and identification were examined using Gram staining and different biochemical tests such as SIM, MRVP, Simmons’ citrate, urease and TSI. *E. coli* standard strain (ATCC 25922) (and *Staphylococcus aureus* standard strain (ATCC 25923) were used as positive and negative control respectively. After verification of the *E. coli* isolates, 2–3 dominant colonies plus all morphologically different colonies (a total of 10 colonies per patient) from each plate of urine (4 colonies) and stool samples (6 colonies) were isolated according to previous studies (18).

### Biochemical fingerprinting using the PhenePlate^TM^ system.

The modified PhenePlate^TM^ (PhP) system for typing of *E. coli* isolates (PhP-RE plate) was used based on the kinetics evaluation of biochemical reactions of each isolate with 12 dehydrated reagents in a 96-well PhP-microplate. The procedure was based on the company’s protocol (5). In summary, all wells in the PhP-plate were filled (0.32 ml into the 1^st^ column, 0.8 ml into all other wells) with suspending medium (1% Peptone + 0.011% BTB + 0.0016M phosphate buffer). Then, using a sterile tooth stick each single bacterial colony from the agar plate was picked and suspended into each well of the 1^st^ inoculation column. The plates were incubated at 37°C in a wet chamber. Each plate was scanned using HP Scanjet 4890 (Hewlett-Packard, USA) after 7, 24 and 48 h. Next, the colors of each well in the scanned plates were analyzed by PhPWIN software.

### Antimicrobial susceptibility test.

Antibiotic susceptibility test for twelve antimicrobial agents was carried out by Kirby-Bauer disc diffusion method (Mast Disc, United Kingdom) on *E. coli* isolates, selected based on biochemical fingerprinting typing, according to the Clinical and Laboratory Standards Institute 2017 guideline (CLSI, 2017), using ampicillin (AP, 10 μg), cefalexin (CFX, 30 μg), trimethoprim-sulfamethoxazole (TS, 25 μg), nalidixic acid (NA, 30 μg), cefixime (CFM, 5 μg), ceftriaxone(CRO, 30 μg), ciprofloxacin (CIP, 5 μg), cefotaxime (CTX, 30 μg), norfloxacin (NOR, 10 μg), gentamicin (GM, 10 μg), imipenem (IMI, 10 μg), and nitrofurantoin (NI, 30 μg).

### DNA extraction.

The DNA was extracted by boiling method. After bacterial passage on the blood agar medium, a single colony was inoculated into 5 ml of Luria-Bertani broth medium and incubated at 37°C on a shaker overnight. Then, 1.5 ml of the medium containing bacteria was centrifuged at 16870 *g* for 5 minutes. In the next step, the supernatant was discarded and the precipitate was suspended with 0.5 ml of sterile distilled water, and boiled at 95°C for 10 minutes. After centrifugation at 16870 *g* for 5 minutes, 2 ml of the supernatant that contained the bacterial DNA was taken, its quantity was measured by BioPhotometer (Eppendorf, Germany) and then used as a template for PCR process (19).

### PCR protocol and phylogenetic grouping.

Multiplex PCR was performed using primers for *chuA* and *yjaA* genes and tspE4.C2 fragment to detect four phylogenetic groups (A, B1, B2 and D) in selected *E. coli* isolates according to the protocol proposed by Clermont et al. (10). Then, *E. coli* isolates were divided into one of the following phylogenetic groups: A (chuA-, yjaA±, tspE4.C2-), B1 (chuA-, yjaA±, tspE4. C2+), B2 (chuA+, yjaA+, tspE4.C2±), and D (chuA+, yjaA-, tspE4.C2±).

### Statistical analysis.

The SPSS software (version 23) was utilized for statistical analysis using Chi-square and Fisher’s exact tests to compare phylogenetic groups and resistance patterns.

## RESULTS

### Clinical data.

A total number of 56 UTI patients (27 acute and 29 recurrent cases) whom UPEC was the causative pathogen were selected. They consisted of both genders (female/male = 3/1) with the mean age of 64 ± 17.5 years. The ratio of acute to recurrent cases in patients under 70 years old was 0.7 (12 *vs.* 17), while in patients in their 7^th^ decade of life was 1 (5 *vs.* 5) and in older patients was 1.4 (10 *vs.* 7). Meanwhile, female patients of this study were mostly in their recurrent phase of UTI (62% of cases); whereas most of the male patients were in their acute phase (79% of cases). Seventy percent of the cases were out-patients. Around 65% (11 out of 17) of in-patients had more than 80 years old. More details and data have been given in Table 1.

**Table 1.** Distribution of patients according to different characteristics

**Category**	**Sub-category**	**No. (%)**
**Phase of UTI**		
Acute		27 (48%)
	Female	16 (59%)
	Male	11 (41%)
	Out-patient	20 (74%)
	In-patient	7 (26%)
	< 70 y	12 (44%)
	70–79 y	5 (19%)
	≥ 80 y	10 (37%)
Recurrent		29 (52%)
	Female	26 (90%)
	Male	3 (10%)
	Out-patient	19 (66%)
	In-patient	10 (34%)
	< 70 y	17 (59%)
	70–79 y	5 (17%)
	≥ 80 y	7 (24%)
**Gender**		
Female		42 (75%)
	Acute	16 (38%)
	Recurrent	26 (62%)
	Out-patient	31 (74%)
	In-patient	11 (26%)
Male		14 (25%)
	Acute	11 (79%)
	Recurrent	3 (21%)
	Out-patient	8 (57%)
	In-patient	6 (43%)
**Age**		
20–39 y		7 (13%)
	Out-patient	5 (71%)
	In-patient	2 (29%)
40–59 y		15 (27%)
	Out-patient	14 (93%)
	In-patient	1 (7%)
60–79 y		17 (30%)
	Out-patient	14 (82%)
	In-patient	3 (18%)
> 80 y		17 (30%)
	Out-patient	6 (35%)
	In-patient	11 (65%)
**In- or out-patients**		
Out-patient		39 (70%)
In-patient		17 (30%)

### Typing by PhenePlate (PhP) biochemical fingerprinting.

**Fig. 1.**
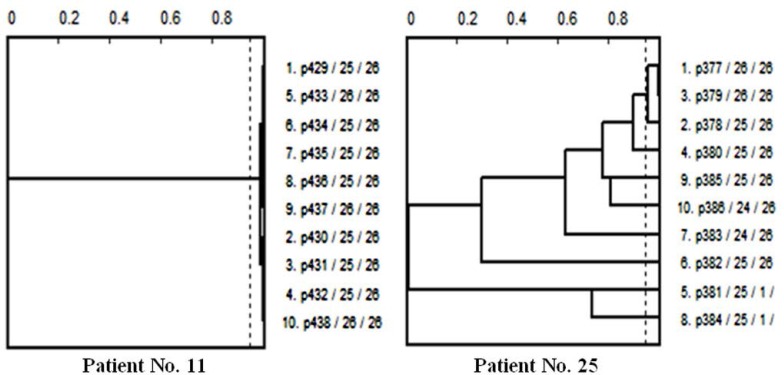
Two dendrograms (as examples) obtained by PhPWIN software for analysis of PhPbiochemical fingerprinting of the isolates of two different patients. The isolates are recovered from either urine or stool samples. In the left dendrogram (Patient No. 11), all the isolates belong to one common type. Therefore one isolate from urine and one from stool were selected. On the other hand, as it has been shown, in Patient No. 25 for instance (right dendrogram), only 3 isolates fit in one common type. Therefore, the rest 7 isolates plus one representative from the common type were selected for next experiments.

As described above, in every patients, 10 colonies were selected (a total number of 560 *E. coli* isolates). All these isolates were evaluated by Phene-Plate (PhP) biochemical fingerprinting. The correlation coefficient score measured as 0.975 using double testing of 4 random samples. Then, using PhPWIN software and based on correlation coefficient score, the isolates were classified into 107 common groups and 96 single types. Among the common groups, 43 groups contained the isolates only from the patients with acute UTI. Twenty-eight groups were only recovered from the patients with recurrent UTI. The other 36 groups were a mix of both acute and recurrent patients. Single types consisted of 53 isolates from the patients with acute UTI and 43 isolates from the ones in recurrent phase. All the isolates which were belonged to the single types and at least one isolate from each common group were selected. Then, the isolates were classified patient by patient using PhPWIN software in correlation coefficient score of 0.95 to add probable missed isolates (at least one isolate from urine and one from stool, Fig. 1). Therefore, 261 out of 560 isolates were finally selected for the next experiments.

### Phylogenetic grouping.

In all 261 selected isolates, the most common phylo-group was B2 (55.2%) followed by A (23.4%). The rests were equally belonged to groups D and B1 (10.7% each, Table 2). This pattern was almost the same in acute and recurrent UTI (data have not been shown). However, in urine samples, where the isolates were expected to be UPEC, phylo-group D came alongside group A as the second common phylo-groups (20.8% each, Table 3). Interestingly, in urine samples of patients with acute UTI, group D was more common than group A (26.8% *vs*. 21.4%), while in the recurrent phase, it was vice versa (14% *vs.* 20%, Table 3). Moreover, among all phylo-groups, it was only group D in which the frequency of the isolates in urine samples of acute UTI was considerably higher not only than stool samples of the same phase (26.8% *vs.* 2.5%), but also than urine samples of recurrent phase (26.8% *vs.* 14%, Table 3). In fact, the percentage of phylo-group D in stool was at the lowest rate (3.9%, Table 3).

**Table 2.** Number and percentage of the isolates of each phylo-group based on different characteristics

**Phylogenetic group**	**Number (%) of isolates**	**UTI Phase**		**Source**		**Gender**		**In-/out-patient**	

		**Acute**	**Recurrent**	**Urine**	**Stool**	**Female**	**Male**	**In-patient**	**Out-patient**
A	61 (23.4%)	30 (49.2%)	31 (50.8%)	22 (36.1%)	39 (63.9%)	48 (78.7%)	13 (21.3%)	15 (24.6%)	46 (75.4%)
B1	28 (10.7%)	11 (39.3%)	17 (60.7%)	10 (35.7 %)	18 (64.3%)	22 (78.6%)	6 (21.4%)	1 (3.6%)	27 (94.4%)
B2	144 (55.2%)	77 (53.5%)	67 (46.5%)	52 (36.1%)	92 (63.9%)	112 (77.8%)	32 (22.2%)	47 (32.6%)	97 (67.4%)
D	28 (10.7%)	17 (60.7%)	11 (39.3%)	22 (78.6%)	6 (21.4%)	14 (50%)	14 (50%)	12 (42.9%)	16 (57.1%)
Total	261 (100%)								

**Table 3.** Distribution of the isolates in urine and stool samples in each phase of UTI based on different phylo-groups

**Source**	**UTI Phase**	**Number (%) of isolates**	**Number (%) of isolates in each phylogenetic group**			

			**A**	**B1**	**B2**	**D**
Urine	Acute	56 (52.8%)	12 (21.4%)	5 (8.9%)	24 (42.9%)	15 (26.8%)
	Recurrent	50 (47.2%)	10 (20%)	5 (10%)	28 (56%)	7 (14%)
	Total	106 (40.6%)	22 (20.8%)	10 (9.4 %)	52 (49%)	22 (20.8%)
Stool	Acute	79 (51%)	18 (22.8%)	6 (7.6%)	53 (67.1%)	2 (2.5%)
	Recurrent	76 (49%)	21 (27.6%)	12 (15.8%)	39 (51.3%)	4 (5.3%)
	Total	155 (59.4%)	39 (25.2%)	18 (11.6%)	92 (59.3%)	6 (3.9%)

Considering phylo-groups, the frequency of the isolates in acute and recurrent UTI patients was similar in phylo-groups B2 and A, while it was notably higher in recurrent than acute phase in group B1. In contrast, in phylo-group D, this frequency was completely opposite (60.7% in acute UTI *vs.* 39.3% in recurrent UTI, Table 2). Meanwhile, when the ratio of the stool’s isolates to urine’s was about 2 to 1 in phylo-groups A, B1 and B2, it was reverse and almost 4 to 1 (78.6% in urine *vs.* 21.4% in stool) in group D. Moreover, while in groups A, B1 and B2, the ratio of the isolates in women to the ones in men was nearly 4 to 1, the frequency of the isolates in phylo-group D was equal in both genders. In the same manner, the percentage of the isolates was much higher in out-patients than in-patients in groups A, B1 and B2, while in phylo-group D, there was no meaningful difference between in- and out-patients (42.9% and 57.1% respectively, Table 2).

### Antibiotic resistance pattern.

**Fig. 2.**
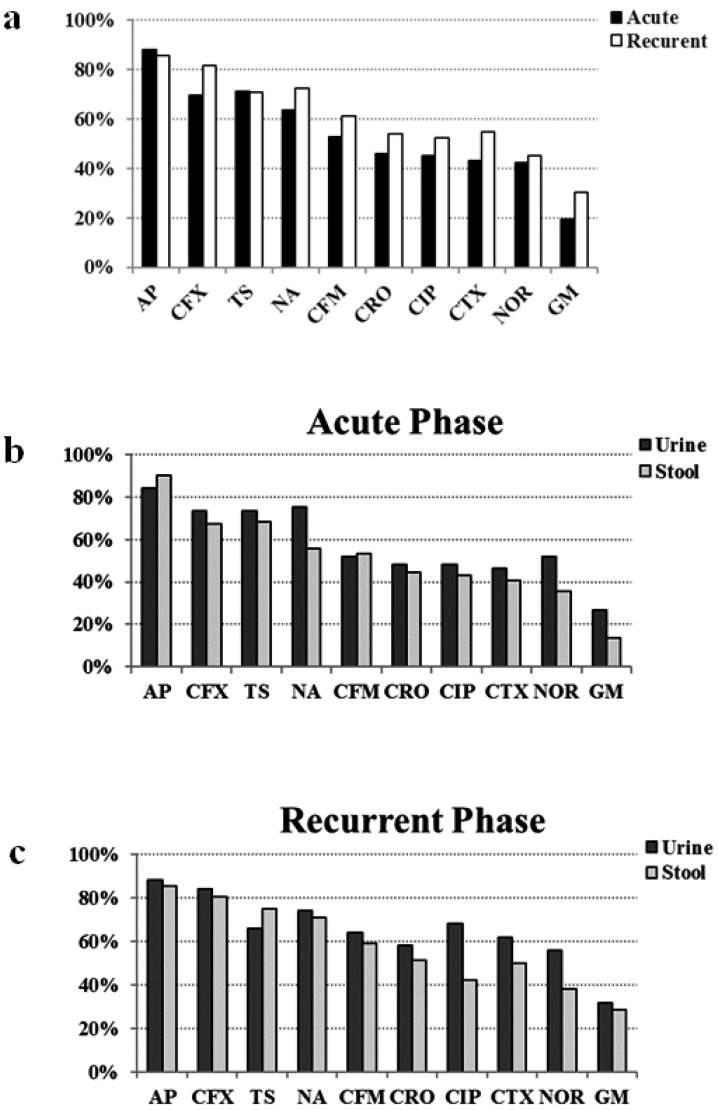
Comparison of antibiotic resistance pattern between acute and recurrent phases of UTI (a). When concerning the pattern based upon acute (b) and recurrent (c) phases, some differences between urine and stool samples are revealed, however the isolates from urine samples show more resistance to most of the antibiotics. AP: ampicillin, CFX: cefalexin, TS: trimethoprim-sulfamethoxazole, NA: nalidixic acid, CFM: cefixime, CRO: ceftriaxone, CIP: ciprofloxacin, CTX: cefotaxime, NOR: norfloxacin, GM: gentamicin.

The 261 selected *E. coli* isolates were completely sensitive to imipenem and nitrofurantoin, and mainly resistant to ampicillin (87%), followed by cefalexin (75.5%), trimethoprim-sulfamethoxazole (70.9%), and nalidixic acid (67.8%). Although nonsignificantly, the isolates from recurrent UTI were more resistant to the most of the antibiotics (exceptions are ampicillin and trimethoprim-sulfamethoxazole) than the ones from acute phase (Fig. 2a). On the other hand, with some exceptions, in both acute and recurrent UTI groups, the isolates obtained from urine were more resistant than the ones from stool (Fig. 2b, c).

**Fig. 3.**
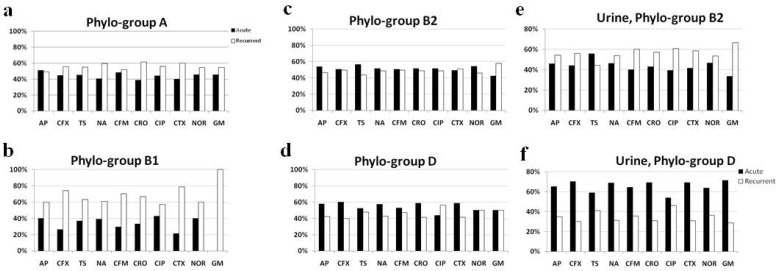
Comparison of antibiotic resistance pattern between acute and recurrent phases in different phylo-groups. While in groups A and B1 the isolates from recurrent UTI are more resistant (a, b), the pattern in groups B2 and D is reverse (c, d). Interestingly, concerning the urine samples of phylo-groups B2 and D, the pattern in group D remains the same while group B2 mimics the pattern of groups B1 and A (e, f). AP: ampicillin, CFX: cefalexin, TS: trimethoprim-sulfamethoxazole, NA: nalidixic acid, CFM: cefixime, CRO: ceftriaxone, CIP: ciprofloxacin, CTX: cefotaxime, NOR: norfloxacinn.

Comparing phylogenetic grouping with antibiotic susceptibility results showed that resistance to the antibiotics was in its highest rate in the isolates belonged to phylo-group D (except to norfloxacin, Table 4). Conversely, the lowest resistance rates were seen in the isolates from group A (except to trimethoprim-sulfamethoxazole, Table 4). By grouping the isolates of each phylo-group based on acute and recurrent phases, it became clear that in group B1, the isolates from recurrent UTI were resistant to all 10 antibiotics notably more than the ones from acute phase (Fig. 3a). In phylo-group A, the pattern was similar (except ampicillin, Fig. 3b). In contract, in groups B2 and D, the isolates were more resistant to the most of the antibiotics in acute UTI than recurrent phase (Fig. 3c, d). However, by narrowing the results to the urine samples, this pattern became reverse in group B2 (except trimethoprim-sulfamethoxazole) while it became clearly more considerable in phylo-group D (Fig. 3e, f).

**Table 4.** Number (and percentage) of antibiotic resistant isolates in each phylo-group

**Phylogenetic group**	**Antibiotics**										**Total**

	**AP**	**CFX**	**TS**	**NA**	**CFM**	**CRO**	**CTX**	**CIP**	**NOR**	**GM**	
A	47 (77%)	38 (69.2%)	42 (68.9%)	37 (60.7%)	29 (47.5%)	26 (42.6%)	25 (41%)	25 (41%)	22 (36%)	11 (18%)	61
B1	25 (89.3%)	23 (82.1%)	19 (67.9%)	18 (64.3%)	17 (60.7%)	15 (53.6%)	14 (50%)	14 (50%)	15 (53.6%)	5 (17.9%)	28
B2	129 (89.6%)	111 (77.1%)	101 (70.1%)	101 (70.1%)	83 (57.6%)	72 (50%)	71 (49.3%)	72 (50%)	63 (43.8%)	38 (26.4%)	144
D	26 (92.9%)	25 (89.3%)	23 (82.1%)	21 (75%)	19 (67.9%)	17 (60.7%)	17 (60.7%)	16 (57.1%)	14 (50%)	10 (34.7%)	28
Total	227 (87%)	197 (75.5%)	185 (70.9%)	177 (67.8%)	148 (56.7%)	130 (49.8%)	127 (48.7%)	127 (48.7%)	114 (43.7%)	64 (24.5%)	261

AP: ampicillin, CFX: cefalexin, TS: trimethoprim-sulfamethoxazole, NA: nalidixic acid, CFM: cefixime, CRO: ceftriaxone, CIP: ciprofloxacin, CTX: cefotaxime, NOR: norfloxacin, GM: gentamicin.

According to previously described definition of multidrug resistant Enterobacteriaceae, 74% of the selected *E. coli* isolates were MDR. The distribution of MDR isolates among acute and recurrent phases was similarly high (70% and 79% respectively). The similar results were also seen in the isolates obtained from urine and stool (76% and 72% respectively). Here again, group D showed the highest MDR pattern among phylo-groups (96% vs. 62% in group A, 68% in group B1 and 76% in group B2). Evaluating the antibiotic resistance patterns based on number of resistance to the antibiotics showed that there were no meaningful differences in the frequency of the isolates either in different UTI phases (acute and recurrent), in different sample sources (urine and stool), or in different phylogenetic groups (data have not been shown).

## DISCUSSION

While B2 and A are the most common phylo-groups among *E. coli* strains in general, extraintestinal pathogenic *E. coli* strains like UPECs usually belong to phylo-groups B2 and D (10). In this study, the general pattern was the same and the most common group among UPECs (urine samples) was also B2 (49% of isolates). However, phylo-groups D and A shared the second place (20.8% each, Table 3). In general, it has been shown that the ecological differences in each geographic region can determine the phylogenetic groups involved in UTIs in each region (20). For example, most studies in France and America have reported that most commensal specimens are related to groups A and B1 (24, 25) while in this study, they mostly belonged to groups B2 and A. This difference in each region can be affected by antibiotics usage or host genetic factors.

In our study, group D showed opposite characteristics compared to other phylo-groups. First, while B2 was the most common phylo-group in both samples as well as both UTI phases, D was the only phylo-group in which the isolates were mostly derived from urine samples (in a ratio of 4 to 1) and patients with acute UTI (in a ratio of 3 to 2, Table 2). In contrast, it was the least common phylo-group in stool and recurrent UTI. Thus, group D seems to be a more specific phylo-group in urine samples, suggesting that isolates of this group might be more urovirulent than others. Moreover, it seems that group D is a more particular phylo-group in patients with acute UTI. Based on previous studies, phylogenetic groups may be one of the effective factors in the onset of infection and isolates causing acute UTI may be more virulent (21, 22). This might be another reason for probably higher virulence rate of *E. coli* isolates in phylo-group D. However, a previous study in China performed by Lou et al. (22) stated that group D isolates were mainly responsible not only for acute UTI, but also for its reinfection; while phylo-group B2 strains were the primarily involved in persistence of recurrent UTI.

Then, the isolates of phylo-group D were distributed equally in both genders and nomeaningful difference in their frequency was seen between in- and out-patients, while in other groups, there was a female-dominant pattern (in a ratio of 4 to 1) and their isolates were mostly belonged to out-patients (Table 2). It has been explained before that due to anatomic and physiologic issues in men, uropathogens need greater virulence to colonize and cause infection in their urinary tract (23). That would probably explain why in this study, the percentage of *E. coli* isolates in men was noticeably higher in group D than in other phylo-groups. Moreover, it has been established so far that the pathogens causing community-acquired infections are less antibiotic resistant than the ones responsible for nosocomial infections, and vice versa (23, 24). Therefore, it would be reasonable to assume that group D might be more virulent than other phylo-groups.

Since *E. coli* strains are dramatically gaining resistance to many antimicrobial agents, antibiotic therapy of UTI has currently become a global problem (14, 25). In this study, there were high resistance rates to some of the antibiotics such as trimethoprim-sulfamethoxazole and fluoroquinolones which are still appropriate in many other countries (26). This could be attributed to their inappropriate use due to their availability and low price, consistent with other studies in Iran (27). Meanwhile, no resistance to imipenem and nitrofurantoin was observed, indicating that the latter and carbapenems as suitable therapeutic regimens for UTI which is in line with other studies (28, 29). Moreover, the *E. coli* isolates were more resistance in patients with recurrent UTI groups, especially in their urine samples. This could be explained by the fact that during antibiotic treatment of acute UTI, the causative UPEC isolates which hide themselves in urothelial cells as well as the intra-intestinal commensal *E. coli* would be exposed to the lower doses of antibiotics. Exposure to the unthreatening doses of the antibiotics would trigger resistome pathways in the isolates which in turn might increase resistance against the antibiotics.

Studies have shown that resistance to antimicrobial agents in *E. coli* is primarily attributed to phylogenetic groups (30). In our study, resistance to the antibiotics was in its highest rate in the isolates belonged to phylo-group D. This is exactly in line with a previous study done by Bashir et al. in Faisalabad region of Pakistan (31). However, in other studies, isolates from group B2 showed the highest antibiotic resistance (16, 32). As it has been previously discussed, the geographic distribution might be a reason for this diversity. On the other hand, group A isolates that mostly represent commensal *E. coli* had the lowest resistance rates which is in contrast to previous studies (30, 31). Moreover, while in commensal phylo-groups (A and B1), the isolates from recurrent UTI were more resistant to antibiotics than the ones from acute phase, in pathogenic phylo-groups (B2 and D) the pattern was reverse, more considerably in group D.

Furthermore, whereas 74% of the all selected isolates were MDR, 76% of isolates from urine samples showed this pattern. A previous study in Bushehr, Iran performed in 2015 by Iranpour et al. (33) reported the rate of MDR isolates as 82% which is near to our result. However, in most neighboring and regional countries higher rates of MDR have been outlined. For instance, in a study by Mukherjee et al. (34) the rate of MDR UPECs in Kolkata, India was detected as high as 92.5%. Besides, in our study, 79% of the isolates recovered from patients with recurrent UTI were MDR, notably higher than the rate (50%) which Foxman et al. reported before (35). It is supposed that MDR pattern mainly occurs due to empiric/inappropriate administration/use of antibiotics. However, significantly more than other phylo-groups, only one isolate in group D was not MDR. In fact, phylo-group D of our study showed a different characteristic in terms of antimicrobial resistance too, compared to other commensal or pathogenic groups.

## CONCLUSION

According to our knowledge, there is no previous epidemiologic study in the region that has collected, at the same time, both urine and stool samples of patients suffering from either acute or recurrent UTI. Based on our results, especially phylogenetic grouping, it could be assumed that UPEC isolates causing acute UTI (which have the ability to break down defensive barriers to penetrate into the urinary tract) are mostly belong to phylo-groups B2 and D. In the meantime, group D isolates seem to be not only more virulent, but also more resistant than other phylo-groups. However, future studies on virulence factors are needed to prove it.
